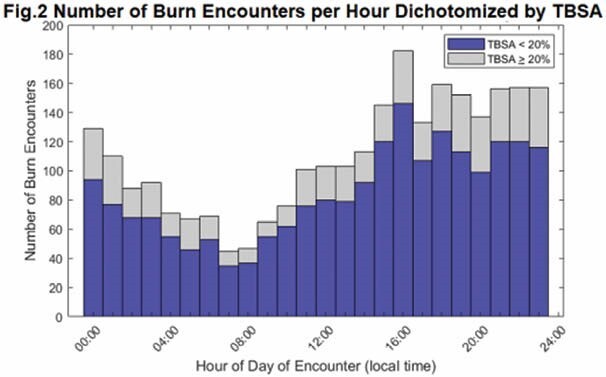# 119 Identifying Temporal Patterns in Burn Admissions

**DOI:** 10.1093/jbcr/irac012.121

**Published:** 2022-03-23

**Authors:** Robel Beyene, David P Stonko, Ronnie Mubang, Stephen Gondek, Jonathan Morrison, Bradley M Dennis

**Affiliations:** Vanderbilt University Medical Center, Nashville, Tennessee; Johns Hopkins Hospital, Manchester, Maryland; Vanderbilt University Medical Center, Nashville, Tennessee; Vanderbilt University Medical Center, Nashville, Tennessee; R Adams Crowley Shock Trauma Center, Baltimore, Maryland; Vanderbilt University Medical Center, Nashville, Tennessee

## Abstract

**Introduction:**

Temporal variations in trauma admissions, based on the time of day, day of week, and day of year, have been previously demonstrated. These variations, which could inform decision making regarding staffing and resource utilization, have not been evaluated with respect to burn admissions. Very little has been published on predicting temporal distribution stratified by total body surface area (TBSA). We hypothesize that temporal patterns exist in the distribution of burn admissions at all TBSA as it relates to time of day, day of week, and day of year.

**Methods:**

This was a cross-sectional observational study of a single burn center over nearly 5 years, from 7/1/2016-3/31/2021, including both pediatric and adult admissions. We captured and plotted bivariate absolute and relative frequency data from all patients who met inclusion criteria in heat-maps showing time of day versus day of week. Frequency analysis was also performed grouped by TBSA against time of day and relative encounters against day of year.

**Results:**

2657 burn patient encounters were analyzed, averaging 1.53 burns per day. Temporal variations were skewed towards evening admission, primarily between 15:00-0:00 hours (p< 0.001). In figure 1, each block of the heatmap represents a one hour block of one day of the week over the nearly five year study period. The color corresponds to the relative frequency of contacts per hour, where 1 represents the mean number of trauma contacts per hour. Evenings (15:00-0:00) have more burn admissions than the rest of the day or night. Figure 2 shows this temporal trend is seen in burn encounters below 20% TBSA as well as those at or greater than 20% TBSA. Unlike trauma admission distribution, which has been shown to increase on weekend, day to day variation does not conform to weekend or weekday distribution. There is no cyclical yearly trend in burn admissions, suggesting that there is no seasonal variation to burn admissions, though individual holidays were not assessed.

**Conclusions:**

We identify temporal variations in burn admissions, including the peak admission window late in the day. However, there is no predictable variation in weekend vs weekday distribution of burns. Furthermore, there is no cyclical annual variation to guide staffing and resource allocation.